# Modeling Alzheimer’s and Other Age Related Human Diseases in Embryonic Systems

**DOI:** 10.3390/jdb6010001

**Published:** 2017-12-22

**Authors:** Chu Hsien Lim, Ajay S. Mathuru

**Affiliations:** 1 Yale NUS College, 16 College Avenue West, Singapore 138610, Singapore; limchuhsien@yale-nus.edu.sg; 2 Institute of Molecular and Cell Biology, 61 Biopolis Drive, Singapore 137673, Singapore

**Keywords:** Alzheimer’s disease, CRY2, *Drosophila melanogaster*, *Caenorhabditis elegans*, *Danio rerio*, optogenetics

## Abstract

Modeling human disease in animals is an important strategy to discover potential methods of intervention. We suggest that there is much to be gained by employing a multi-model approach that takes advantage of different animal systems used in the laboratory simultaneously. We use the example of modeling Alzheimer’s disease in *Drosophila melanogaster*, *Caenorhabditis elegans*, and *Danio rerio* to illustrate how such an approach can be employed to investigate the pathophysiology of the disease.

## 1. Introduction

Animal models are indispensable tools for understanding biology and for designing strategies to mitigate human disease. An important question to address then is the choice of the animal species when modeling a disorder or a disease. After all, animals may not suffer from the same ailments as humans do. A popular idea in the field of neuroethology is to study a ‘champion’ species or a specialist in the particular motor or sensory function of interest [1,2,3]. An echo-locating bat may reveal the workings of auditory processing or a cephalopod may help us identify a neural mechanism for camouflage. Indeed, unusual insights into cancer pathology have come to us via naked mole rats that are highly resistant to cancers in spite of a life spanning around three decades [4,5]. However, this is not entirely suitable to tease apart the molecular and cellular processes underlying disease progression in many cases that often require extensive experimentation and elaborate screens. 

Historically, rodents and non-human primates have received the most attention in medicine as mammalian models, particularly in the final phases of drug or vaccine development prior to clinical trials in humans. However, the costs and infrastructure associated with running such experiments at scale are fast becoming prohibitive. Instead *Drosophila melanogaster*, *Caenorhabditis elegans*, and *Danio rerio* (colloquially, fruit flies, nematode worms, and zebrafish) are increasingly used to study various aspects of diseases for exactly the same reasons that made them popular for studying fundamental biology—the ease of rearing them in the lab, performing genetic manipulations, molecular and biochemical analyses, and behavioral studies. For instance, fruit fly, worm, and zebrafish models have been critical to elucidate the RAS signaling pathway in cancer, polyglutamine-expansion disorders, and melanoma respectively among other diseases [6,7,8,9,10]. We suggest that using all three in combination may circumvent some of the challenges posed in the search of a ‘champion’ animal model that satisfactorily recapitulates a disease and allows studies of genetics, physiology, and behavior. In addition, the tools available for studying development in these systems become highly advantageous for dissociating incidental age-related changes from changes causally linked in an age dependent disease.

## 2. Alzheimer’s Disease: An Unresolved Problem

In this commentary, we turn our attention to Alzheimer’s disease (AD). AD is the most common cause of dementia, and a debilitating age-associated neurodegenerative disease currently lacking effective treatment [11,12]. According to a non-profit, voluntary organization specializing in Alzheimer’s care, AD is the sixth leading cause of death in America, and there are now 5.5 million Americans living with it [13]. Globally, there are 46.8 million people living with dementia, including AD and this number is expected to increase to reach 131.5 million people by 2050 [14]. Global burden of disease studies suggest that the cost of AD and other dementia-related illness is in the range of USD 250 billion in America, and over USD 600 billion globally [13,15]. Such statistics highlight the prevalence of AD and other dementia-related illnesses and the grave public health challenge that future generations face. 

Even after several decades of research, the exact cause of AD is yet to be clearly established. However, two classic neuropathological signs are associated with the disease. Namely, neurofibrillary tangles and extracellular Amyloid-β (Aβ) plaques in the human brain [16,17]. Tangles are thought to be caused by misfolding and hyperphosphorylation of tau proteins normally involved in microtubule stabilization [17]. Plaques, on the other hand, are aggregates of the beta-amyloid peptide—a cleavage product of a protein that is normally expressed throughout the ubiquitously called the amyloid precursor protein (APP) [18]. 

While both these processes are associated with AD, here, for the purpose of illustration, we only consider the amyloid hypothesis. According to this hypothesis, the accumulation of Aβ in various forms, as plaques or as soluble oligomers cause neuronal damage and the consequent inflammatory response that eventually leads to the deleterious symptoms associated with AD [19]. Evidence underpinning this association comes from familial cases of early-onset AD, which is linked to germline mutations in APP [20] and the presenilin genes involved in cleaving Aβ from APP [21]. The amyloid hypothesis proponents use pathological similarities between familial and sporadic AD as an argument, as well as observations of other age-related diseases such as the myositis-like phenotype caused by Aβ accumulation in transgenic mice overexpressing Aβ in muscle tissue [22]. 

Despite such evidence, the pathophysiology of Aβ plaques and their relation to AD is not a settled issue. Critics of the amyloid hypothesis point out findings of Aβ plaques in brains of individuals who did not show any symptoms of AD at the time of death. Other unresolved issues including poor correlation between plaque load and cognitive scores and heterogeneity in pathology have called into question the causal association ascribed to Aβ plaques [23]. There has been a shift of focus from plaques to the other potential malfunctions including to role of soluble forms of Aβ oligomerisation that further triggers a downstream cascade [24]. A recent paper also demonstrated that blood-derived Aβ resulted in pathogenic symptoms of AD in mice models, suggesting that Aβ plaques need not originate in the brain to evoke pathological symptoms [25]. Given the current status, whether extracellular Aβ plaques have a detrimental role in AD, or if they have a protective role before other malfunctions accumulate continues to be a central question [26]. Further, even though extracellular plaques are considered a hallmark of AD, consensus on whether problems arise due to Aβ aggregates formed intracellularly in neurons, or from secreted peptides extracellulary (or either), is also lacking [27,28,29].

## 3. Combining Forces

To unravel these questions, one approach could be to first clearly evaluate if Aβ plaque formation has any impact on neuronal health and cognitive performance. One approach is to use a multi-model study design that capitalizes on the unique advantages of flies, nematodes, and zebrafish.

Research using *Drosophila* has been vital to dissect genetic pathways critical for the formation, migration, and activity of Aβ plaques [6]. Using the galactose-inducible yeast system, commonly known as the Gal4/UAS system [30], transgenic flies can be easily generated to analyze several genetic pathways in a short period. The genetic pathways identified from such analyses can be further investigated in *C. elegans* biochemically. *C. elegans* has been widely employed to investigate the effects of aging and various neurodegenerative diseases, including AD [7]. With a short life cycle of three days from egg to adult, they are highly amenable to evaluate the “druggability” of targets among the biochemical pathways [31]. Feeding assays can be designed to implement large-scale drug screens or screens based on RNA interference (RNAi) knockdown [7]. Another factor when modeling human pathologies is to recapitulate the biological outcomes described in humans. Compared to the two invertebrate animal models, the vertebrate nervous system of zebrafish is more appropriate to study the consequences of Aβ plaques in the brain. Due to their transparent nature, zebrafish are also an excellent tool to investigate neural activity in vivo, thereby making them particularly useful when studying neurophysiology. Using whole brain imaging and cell-type specific labeling techniques, unique neurons in any region of the brain can be studied with ease [32]. In addition, several behavioral paradigms have already been developed that can be employed to evaluate the cognitive impact if neurodegeneration is occurring [33].

Advancements in imaging and genetic techniques further enhance the utility of this multi-model approach. One such technique is optogenetics—an extremely versatile tool using light to examine physiological processes [34]. Compared to the constraints posed by electrical recordings and pharmacological interventions in the past, optogenetics can be used to examine specific cellular activities with extremely high precision and speed in vivo. Recently, cryptochrome2 (CRY2) protein system from the *Arabidopsis* was modified as an optogenetic tool to study the control of cortical actin dynamics in cell culture and cell contractility during tissue morphogenesis in *Drosophila* embryos [35,36]. Activation of the CRY2 oligomerization at 488 nm and inhibition in the dark allows researchers to manipulate protein–protein interactions without the need for other external agents [37]. 

**Figure 1:**
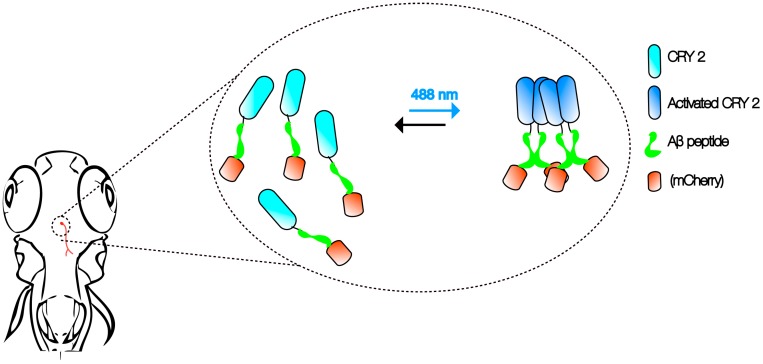
Aβ CRY2 optogenetic system. The schematic shows an example of in vivo, light-dependent, oligomerization switch for the formation or dissolution of Aβ aggregates in region of interest (neurons) in a transgenic zebrafish.

It is not hard to imagine that the same CRY2 optogenetic system can be employed with Aβ to create an in vivo, light-dependent, oligomerization switch for the formation or dissolution of Aβ aggregates (Figure 1). By generating transgenics in all three organisms in the neurons, or cells of interest, the spatiotemporal kinetics of Aβ plaque formation can be controlled at will to address the question of their relationship and AD pathogenesis. Light-inducible aggregates in worms will allow fast screening for drugs that are effective in dissolution when administered before, or after, or when plaques form repeatedly. Transparency of zebrafish allows imaging the kinetics of plaque formation. We envisage that the use of such a technique in fish will also shed light on the link between intracellular Aβ peptide aggregates and extracellular plaques. In addition, promising drugs screened in the worm studies can be rapidly evaluated in the context of a vertebrate brain. A battery of behavioral assays reporting on learning, memory, anxiety, and depression can then be employed to assess the impact [33]. Finally, flies that express Aβ CRY2 in specific organs, at specific times, will be highly informative to understand the spatiotemporal dynamics of plaque location for any pathogenic effect to be visible, as well as to identify genetic interactors at different stages in case pathogenicity is observed [25]. Together, these will likely provide firm answers to the questions raised by the amyloid hypothesis of AD, especially on the role of soluble vs. Aβ plaques as plaque formation can be engineered externally.

The potential of using such a combinatory approach of various model organisms and optogenetics is therefore vast, can be applied to other diseases and will only continue to expand as further improvements in technology become available.
